# (1*RS*,2*SR*,5*SR*)-9-Benzyl-2-[(1*RS*)-1-hy­droxy­benz­yl]-9-aza­bicyclo­[3.3.1]nonan-3-one from synchrotron data

**DOI:** 10.1107/S1600536812014754

**Published:** 2012-04-13

**Authors:** Ryszard Lazny, Karol Wolosewicz, Zbigniew Dauter, Krzysztof Brzezinski

**Affiliations:** a Institute of Chemistry, University of Bialystok, Hurtowa 1, 15-399 Bialystok, Poland; b Synchrotron Radiation Research Section, MCL, National Cancer Institute, Argonne National Laboratory, Biosciences Division, Bldg 202, Argonne, IL 60439, USA

## Abstract

In the crystal structure of the racemic title compound, C_22_H_25_NO_2_, solved and refined against sychrotron diffraction data, the hy­droxy group and the carbonyl O atom participate in the formation of O—H⋯O hydrogen bonds between pairs of enanti­omers related by a crystallographic centre of symmetry.

## Related literature

For recent background literature on the synthesis, structure and applications of related granatane-derived aldols, see: Lazny *et al.* (2011*a* ▶) and references cited therein. For the stereoselective syntheses, applications and structures of related tropinone aldols, see: Sienkiewicz *et al.* (2009 ▶); Lazny *et al.* (2011*b* ▶); Brzezinski *et al.* (2012 ▶) and for related nortropin­one aldols, see: Lazny *et al.* (2001 ▶, 2010 ▶); Lazny & Nodzewska (2003 ▶).
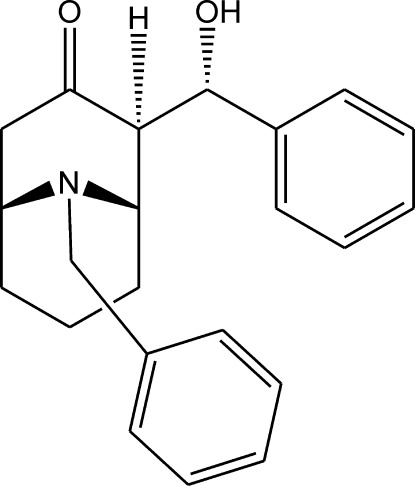


## Experimental

### 

#### Crystal data

C_22_H_25_NO_2_
*M* *_r_* = 335.43
Monoclinic, 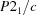

*a* = 14.380 (3) Å
*b* = 9.3100 (19) Å
*c* = 13.270 (3) Å
β = 106.21 (3)°
*V* = 1705.9 (6) Å^3^
*Z* = 4
Synchrotron radiation
λ = 0.61992 Å
μ = 0.08 mm^−1^
*T* = 100 K
0.3 × 0.1 × 0.1 mm

#### Data collection

Mar Research MAR315 CCD diffractometer
Absorption correction: multi-scan (*SCALEPACK*; Otwinowski & Minor, 2003 ▶) *T*_min_ = 0.975, *T*_max_ = 0.992
64629 measured reflections
8582 independent reflections
7757 reflections with *I* > 2σ(*I*)
*R*_int_ = 0.046

#### Refinement

*R*[*F* ^2^ > 2σ(*F* ^2^)] = 0.040
*wR*(*F* ^2^) = 0.119
*S* = 1.03
8582 reflections
227 parameters
H-atom parameters constrained
Δρ_max_ = 0.58 e Å^−3^
Δρ_min_ = −0.30 e Å^−3^

### 

Data collection: *NECAT APS *beamline software; cell refinement: *HKL-2000* (Otwinowski & Minor, 1997 ▶); data reduction: *HKL-2000*; program(s) used to solve structure: *SHELXD* (Sheldrick, 2008 ▶); program(s) used to refine structure: *SHELXL97* (Sheldrick, 2008 ▶); molecular graphics: *ORTEP-3* (Farrugia, 1997 ▶) and *pyMOL* (DeLano, 2002 ▶); software used to prepare material for publication: *SHELXL97*.

## Supplementary Material

Crystal structure: contains datablock(s) global, I. DOI: 10.1107/S1600536812014754/kp2402sup1.cif

Structure factors: contains datablock(s) I. DOI: 10.1107/S1600536812014754/kp2402Isup2.hkl

Supplementary material file. DOI: 10.1107/S1600536812014754/kp2402Isup3.cml

Additional supplementary materials:  crystallographic information; 3D view; checkCIF report

## Figures and Tables

**Table 1:** Hydrogen-bond geometry (Å, °)

*D*—H⋯*A*	*D*—H	H⋯*A*	*D*⋯*A*	*D*—H⋯*A*
O10—H10*A*⋯O3^i^	0.84	2.11	2.9298 (9)	165

Symmetry code: (i) 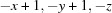.

**Fig. 1.**
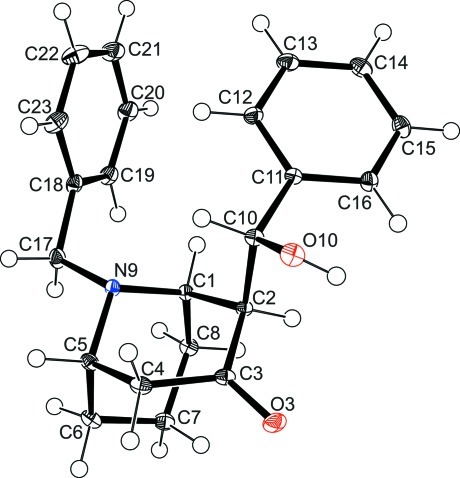
The molecular structure of the title compound. Displacement ellipsoids are drawn at the 50% probability level.

**Fig. 2.**
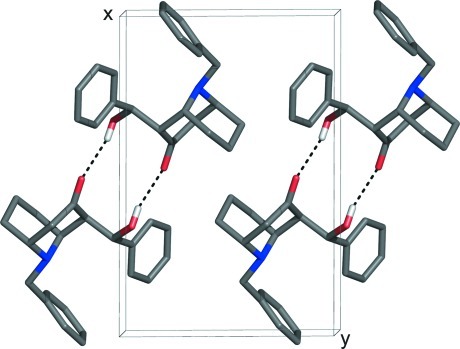
Crystal packing viewed along z-axis. Dashed lines represent hydrogen bonds. For clarity, only hydrogen atoms involved in the intermolecular interactions are shown.

## supplementary crystallographic information

### Comment

Granatane (9-methyl-9-azabicyclo[3.3.1]nonane) and norgranatane (9-azabicyclo[3.3.1]nonane) are known scaffolds of several molecules tested *e.g.* as antagonists of human serotonin type-3 receptor (5-HT3*R*). So far, relatively few synthetic and natural granatane derivatives have been synthesized and studied as potential pharmaceutically useful agents. Related diastereomerically and enantimerically pure aldols of granatanone have been recently described (Lazny *et al.*, 2011*a*). Related aldols of tropinone have been used as key intermediates in several stereoselective syntheses of alkaloids *e.g.*, ferrugine (Sienkiewicz *et al.*, 2009 and references cited therein). The *N*-benzyl derivative is a potentially useful intermediate for synthesis of nor-aldols of granatanone, preparation of which is unknown. Effective, stereoselective syntheses of related nortropinone aldols (Lazny & Nodzewska, 2003; Lazny *et al.*, 2001) is still an unsolved problem. Therefore synthetically equivalent *N*-benzylnorgranatanone aldols should open a route to preparative accessibility of substituted norgranatanes for biomedical studies. The described *N*-benzyl derivative was prepared by a procedure analogous to methods known for *N*-methyl aldols. The synthetic procedure gave a racemic product.

The crystal structure of the title compound contains one molecule in the asymmetric unit (Fig. 1). Two intermolecular hydrogen bonds are formed between a pair of enantiomers in the crystal lattice. hydroxy group and carbonyl oxygen atom of the azabicyclo[3.3.1]nonan-3-one system participate in this interaction (Table 1, Fig. 2).

### Experimental

A solution of n-butyllithium in hexane (2.5*M*, 0.88 mL, 2.0 mmol) was added dropwise to a cooled (273 K) and stirred solution of diisopropylamine (0.3 ml, 2.2 mmol) in tetrahydrofuran (6 mL). The mixture was stirred for 30 min at 273 K, and cooled down to 195 K. Then a solution of *N*-benzylnorgranatanone (0.459 g, 2.0 mmol) in tetrahydrofuran (3 mL) was added dropwise. After stirring for 90 min, benzaldehyde (0.22 ml, 2.18 mmol) was added dropwise and the mixture was stirred for another 15 min. The reaction was quenched with saturated aq. NH_4_Cl (2 mL), the mixture was diluted with water (10 mL), and extracted with dichloromethane (3 × 20 mL). The combined organic extracts were dried over Na_2_SO_4_ and concentrated to give the crude product as a white solid (0.663 g, 99%). Crystallization from a mixed solvent system heptane/dichloromethane gave the product (0.243 g, 75%) as white crystals. Analytical sample was recrystallized from ethyl acetate. [m.p. 412–413 K, *R_f_*: 0.65 (50% ethyl acetate/hexanes); HR (MS-ESI): MNa+, found 358.1794, C_22_H_25_NNaO_2_ requires 358.1783; ^1^H NMR (CDCl_3_): 7.43–7.41 (m, 4H), 7.40–7.34 (m, 1H), 7.31–7.28 (m, 1H), 7.26–7.20 (m, 3H), 6.67 (s, 1H), 5.16 (d, *J*= 4.0 Hz, 1H), 4.04 (q, *J*= 12.8 Hz, 2H), 3.41 (d, *J*= 3.6 Hz, 1H), 3.68–3.64 (m, 1H), 2.92 (dd, *J_1_*= 16.2 Hz, *J_2_*=7.0 Hz, 1H), 2.57 (d, *J*= 4.0 Hz, 1H), 2.43 (d, *J*= 16.2 Hz, 1H), 2.19–2.13 (m, 2H), 1.65–1.62 (m, 2H), 1.37–1.32 (m, 2H)].

### Refinement

All hydrogen atoms were constrained to idealized positions with C—H distances fixed at 0.95–1.00 Å and O—H distances fixed at 0.84 Å and *U*_iso_(H) = 1.5*U*_eq_(C) for hydroxy hydrogen atom and 1.2*U*_eq_(C) for others.

### Crystal data

**Table:**

C_22_H_25_NO_2_	*F*(000) = 720
*M**_r_* = 335.43	*D*_x_ = 1.306 Mg m^−^^3^
Monoclinic, *P*2_1_/*c*	Synchrotron radiation, λ = 0.61992 Å
Hall symbol: -P 2ybc	Cell parameters from 8582 reflections
*a* = 14.380 (3) Å	θ = 2.4–31.7°
*b* = 9.3100 (19) Å	µ = 0.08 mm^−^^1^
*c* = 13.270 (3) Å	*T* = 100 K
β = 106.21 (3)°	Needle, colourless
*V* = 1705.9 (6) Å^3^	0.3 × 0.1 × 0.1 mm
*Z* = 4	

### Data collection

**Table:**

Mar Research MAR315 CCD diffractometer	8582 independent reflections
Radiation source: NECAT 24ID-C synchrotron beamline APS, USA	7757 reflections with *I* > 2σ(*I*)
Si111 double crystal monochromator	*R*_int_ = 0.046
ω scans	θ_max_ = 31.7°, θ_min_ = 2.4°
Absorption correction: multi-scan (*SCALEPACK*; Otwinowski *et al.*, 2003)	*h* = −24→23
*T*_min_ = 0.975, *T*_max_ = 0.992	*k* = −15→0
64629 measured reflections	*l* = 0→22

### Refinement

**Table:**

Refinement on *F*^2^	Primary atom site location: structure-invariant direct methods
Least-squares matrix: full	Secondary atom site location: difference Fourier map
*R*[*F*^2^ > 2σ(*F*^2^)] = 0.040	Hydrogen site location: inferred from neighbouring sites
*wR*(*F*^2^) = 0.119	H-atom parameters constrained
*S* = 1.03	*w* = 1/[σ^2^(*F*_o_^2^) + (0.076*P*)^2^ + 0.3*P*] where *P* = (*F*_o_^2^ + 2*F*_c_^2^)/3
8582 reflections	(Δ/σ)_max_ = 0.001
227 parameters	Δρ_max_ = 0.58 e Å^−^^3^
0 restraints	Δρ_min_ = −0.30 e Å^−^^3^

### Special details

**Table:**

Experimental. The crystal was mounted with vaseline on a pin-attached capillary. Upon mounting, the crystal was quenched to 100 K in a nitrogen-gas stream supplied by an Oxford Cryo-Jet. Diffraction data were measured at the station 24-ID—C of the APS synchrotron by rotation method.
Geometry. All e.s.d.'s are estimated using the full covariance matrix. The cell e.s.d.'s are taken into account individually in the estimation of e.s.d.'s in distances, angles and torsion angles; correlations between e.s.d.'s in cell parameters are only used when they are defined by crystal symmetry.
Refinement. Refinement of *F*^2^ against all reflections. The weighted *R*-factor *wR* and goodness of fit *S* are based on *F*^2^, conventional *R*-factors *R* are based on *F*, with *F* set to zero for negative *F*^2^. The threshold expression of *F*^2^ > 2σ(*F*^2^) is used only for calculating *R*-factors *etc*. and is not relevant to the choice of reflections for refinement. *R*-factors based on *F*^2^ are statistically about twice as large as those based on *F*.

### Fractional atomic coordinates and isotropic or equivalent isotropic displacement parameters (Å^2^)

**Table:**

	*x*	*y*	*z*	*U*_iso_*/*U*_eq_	
C1	0.30958 (3)	0.78442 (5)	0.11193 (4)	0.00793 (8)	
H1	0.2751	0.7328	0.1570	0.010*	
C2	0.36548 (3)	0.67096 (5)	0.06626 (4)	0.00840 (8)	
H2	0.4214	0.6355	0.1241	0.010*	
C3	0.40394 (4)	0.73073 (6)	−0.02043 (4)	0.01010 (8)	
O3	0.48222 (3)	0.69336 (5)	−0.03110 (4)	0.01510 (8)	
C4	0.33793 (4)	0.83261 (6)	−0.09524 (4)	0.01142 (9)	
H4A	0.2903	0.7762	−0.1489	0.014*	
H4B	0.3768	0.8897	−0.1317	0.014*	
C5	0.28317 (4)	0.93572 (6)	−0.04151 (4)	0.01032 (8)	
H5	0.2313	0.9837	−0.0976	0.012*	
C6	0.34966 (4)	1.05349 (6)	0.02088 (4)	0.01288 (9)	
H6A	0.3832	1.1020	−0.0254	0.015*	
H6B	0.3099	1.1261	0.0443	0.015*	
C7	0.42514 (4)	0.99255 (6)	0.11677 (4)	0.01233 (9)	
H7A	0.4588	1.0726	0.1614	0.015*	
H7B	0.4740	0.9370	0.0933	0.015*	
C8	0.37747 (4)	0.89508 (6)	0.18111 (4)	0.01071 (8)	
H8A	0.3402	0.9548	0.2178	0.013*	
H8B	0.4284	0.8442	0.2350	0.013*	
N9	0.23546 (3)	0.85037 (5)	0.02376 (3)	0.00863 (7)	
C10	0.29832 (3)	0.54154 (6)	0.02123 (4)	0.00928 (8)	
H10	0.2345	0.5823	−0.0194	0.011*	
O10	0.33364 (3)	0.45861 (5)	−0.05034 (3)	0.01360 (8)	
H10A	0.3898	0.4294	−0.0203	0.020*	
C11	0.27972 (3)	0.45235 (5)	0.10896 (4)	0.00883 (8)	
C12	0.18961 (4)	0.45726 (6)	0.12906 (4)	0.01206 (9)	
H12	0.1403	0.5180	0.0881	0.014*	
C13	0.17105 (4)	0.37413 (7)	0.20856 (5)	0.01465 (10)	
H13	0.1093	0.3782	0.2213	0.018*	
C14	0.24272 (4)	0.28511 (6)	0.26930 (4)	0.01428 (10)	
H14	0.2298	0.2270	0.3227	0.017*	
C15	0.33362 (4)	0.28183 (6)	0.25111 (4)	0.01342 (9)	
H15	0.3833	0.2228	0.2933	0.016*	
C16	0.35203 (4)	0.36467 (6)	0.17144 (4)	0.01158 (9)	
H16	0.4141	0.3616	0.1595	0.014*	
C17	0.16235 (4)	0.93738 (6)	0.05545 (4)	0.01253 (9)	
H17A	0.1176	0.9806	−0.0079	0.015*	
H17B	0.1954	1.0166	0.1014	0.015*	
C18	0.10470 (4)	0.85031 (6)	0.11268 (4)	0.01131 (9)	
C19	0.13134 (4)	0.84836 (6)	0.22226 (4)	0.01282 (9)	
H19	0.1831	0.9076	0.2603	0.015*	
C20	0.08325 (4)	0.76093 (7)	0.27672 (5)	0.01567 (10)	
H20	0.1035	0.7585	0.3512	0.019*	
C21	0.00550 (4)	0.67733 (7)	0.22170 (5)	0.01794 (11)	
H21	−0.0269	0.6164	0.2584	0.022*	
C22	−0.02467 (4)	0.68332 (8)	0.11257 (5)	0.01934 (11)	
H22	−0.0794	0.6292	0.0749	0.023*	
C23	0.02508 (4)	0.76839 (7)	0.05837 (5)	0.01621 (10)	
H23	0.0047	0.7707	−0.0162	0.019*	

### Atomic displacement parameters (Å^2^)

**Table:**

	*U*^11^	*U*^22^	*U*^33^	*U*^12^	*U*^13^	*U*^23^
C1	0.00930 (16)	0.00893 (19)	0.00596 (16)	0.00061 (13)	0.00280 (13)	−0.00010 (13)
C2	0.00893 (16)	0.00910 (19)	0.00783 (16)	−0.00015 (13)	0.00343 (13)	−0.00046 (13)
C3	0.01167 (18)	0.0101 (2)	0.01030 (18)	−0.00211 (14)	0.00599 (14)	−0.00254 (14)
O3	0.01403 (16)	0.01532 (19)	0.01972 (19)	0.00038 (13)	0.01092 (14)	−0.00186 (15)
C4	0.01423 (19)	0.0137 (2)	0.00782 (17)	−0.00160 (15)	0.00560 (15)	0.00005 (15)
C5	0.01295 (18)	0.0109 (2)	0.00784 (17)	−0.00003 (15)	0.00418 (14)	0.00183 (14)
C6	0.0171 (2)	0.0097 (2)	0.0129 (2)	−0.00171 (16)	0.00595 (16)	0.00079 (15)
C7	0.01364 (19)	0.0116 (2)	0.01232 (19)	−0.00284 (15)	0.00451 (15)	−0.00222 (16)
C8	0.01263 (18)	0.0115 (2)	0.00778 (17)	−0.00095 (15)	0.00251 (14)	−0.00161 (14)
N9	0.00942 (15)	0.01032 (18)	0.00683 (15)	0.00179 (12)	0.00338 (12)	0.00153 (12)
C10	0.01063 (17)	0.0100 (2)	0.00774 (17)	−0.00114 (14)	0.00340 (13)	−0.00120 (14)
O10	0.01844 (17)	0.01365 (18)	0.01040 (15)	−0.00106 (13)	0.00681 (13)	−0.00429 (13)
C11	0.00996 (17)	0.00859 (19)	0.00832 (17)	−0.00088 (13)	0.00316 (13)	−0.00087 (13)
C12	0.01043 (18)	0.0135 (2)	0.0130 (2)	−0.00065 (15)	0.00445 (15)	0.00118 (16)
C13	0.0149 (2)	0.0158 (2)	0.0156 (2)	−0.00242 (17)	0.00802 (17)	0.00100 (18)
C14	0.0206 (2)	0.0125 (2)	0.01139 (19)	−0.00221 (17)	0.00719 (17)	0.00027 (16)
C15	0.0181 (2)	0.0119 (2)	0.01043 (19)	0.00227 (16)	0.00425 (16)	0.00130 (16)
C16	0.01218 (18)	0.0124 (2)	0.01057 (18)	0.00178 (15)	0.00386 (14)	0.00047 (15)
C17	0.01361 (19)	0.0129 (2)	0.0126 (2)	0.00440 (16)	0.00617 (15)	0.00246 (16)
C18	0.01020 (17)	0.0139 (2)	0.01086 (18)	0.00307 (15)	0.00472 (14)	0.00007 (15)
C19	0.01191 (18)	0.0166 (2)	0.01089 (19)	0.00131 (16)	0.00477 (15)	−0.00027 (16)
C20	0.0147 (2)	0.0212 (3)	0.0132 (2)	0.00182 (18)	0.00730 (17)	0.00159 (18)
C21	0.0158 (2)	0.0205 (3)	0.0214 (3)	−0.00107 (19)	0.01156 (19)	−0.0008 (2)
C22	0.0138 (2)	0.0253 (3)	0.0208 (3)	−0.00457 (19)	0.00800 (19)	−0.0067 (2)
C23	0.01207 (19)	0.0241 (3)	0.0130 (2)	−0.00040 (18)	0.00449 (16)	−0.00376 (19)

### Geometric parameters (Å, º)

**Table:**

C1—N9	1.4784 (8)	O10—H10A	0.8400
C1—C8	1.5353 (8)	C11—C12	1.3947 (8)
C1—C2	1.5496 (7)	C11—C16	1.3977 (8)
C1—H1	1.0000	C12—C13	1.3931 (8)
C2—C3	1.5147 (7)	C12—H12	0.9500
C2—C10	1.5554 (8)	C13—C14	1.3910 (9)
C2—H2	1.0000	C13—H13	0.9500
C3—O3	1.2236 (7)	C14—C15	1.3942 (9)
C3—C4	1.5033 (8)	C14—H14	0.9500
C4—C5	1.5374 (8)	C15—C16	1.3924 (8)
C4—H4A	0.9900	C15—H15	0.9500
C4—H4B	0.9900	C16—H16	0.9500
C5—N9	1.4778 (7)	C17—C18	1.5075 (8)
C5—C6	1.5362 (8)	C17—H17A	0.9900
C5—H5	1.0000	C17—H17B	0.9900
C6—C7	1.5324 (9)	C18—C23	1.3969 (9)
C6—H6A	0.9900	C18—C19	1.3966 (8)
C6—H6B	0.9900	C19—C20	1.3936 (8)
C7—C8	1.5326 (8)	C19—H19	0.9500
C7—H7A	0.9900	C20—C21	1.3903 (10)
C7—H7B	0.9900	C20—H20	0.9500
C8—H8A	0.9900	C21—C22	1.3920 (10)
C8—H8B	0.9900	C21—H21	0.9500
N9—C17	1.4782 (7)	C22—C23	1.3941 (9)
C10—O10	1.4232 (7)	C22—H22	0.9500
C10—C11	1.5133 (7)	C23—H23	0.9500
C10—H10	1.0000		
			
N9—C1—C8	113.09 (5)	O10—C10—C2	112.19 (4)
N9—C1—C2	108.16 (4)	C11—C10—C2	110.71 (4)
C8—C1—C2	112.25 (4)	O10—C10—H10	106.9
N9—C1—H1	107.7	C11—C10—H10	106.9
C8—C1—H1	107.7	C2—C10—H10	106.9
C2—C1—H1	107.7	C10—O10—H10A	109.5
C3—C2—C1	112.69 (4)	C12—C11—C16	118.86 (5)
C3—C2—C10	108.18 (4)	C12—C11—C10	120.15 (5)
C1—C2—C10	110.12 (4)	C16—C11—C10	120.99 (4)
C3—C2—H2	108.6	C13—C12—C11	120.75 (5)
C1—C2—H2	108.6	C13—C12—H12	119.6
C10—C2—H2	108.6	C11—C12—H12	119.6
O3—C3—C4	122.33 (5)	C14—C13—C12	120.14 (5)
O3—C3—C2	121.74 (5)	C14—C13—H13	119.9
C4—C3—C2	115.87 (4)	C12—C13—H13	119.9
C3—C4—C5	113.48 (4)	C13—C14—C15	119.45 (5)
C3—C4—H4A	108.9	C13—C14—H14	120.3
C5—C4—H4A	108.9	C15—C14—H14	120.3
C3—C4—H4B	108.9	C16—C15—C14	120.36 (5)
C5—C4—H4B	108.9	C16—C15—H15	119.8
H4A—C4—H4B	107.7	C14—C15—H15	119.8
N9—C5—C6	112.88 (4)	C15—C16—C11	120.42 (5)
N9—C5—C4	108.53 (5)	C15—C16—H16	119.8
C6—C5—C4	111.92 (4)	C11—C16—H16	119.8
N9—C5—H5	107.8	N9—C17—C18	112.54 (5)
C6—C5—H5	107.8	N9—C17—H17A	109.1
C4—C5—H5	107.8	C18—C17—H17A	109.1
C7—C6—C5	111.92 (5)	N9—C17—H17B	109.1
C7—C6—H6A	109.2	C18—C17—H17B	109.1
C5—C6—H6A	109.2	H17A—C17—H17B	107.8
C7—C6—H6B	109.2	C23—C18—C19	118.50 (5)
C5—C6—H6B	109.2	C23—C18—C17	121.36 (5)
H6A—C6—H6B	107.9	C19—C18—C17	120.13 (5)
C6—C7—C8	111.01 (4)	C20—C19—C18	121.06 (6)
C6—C7—H7A	109.4	C20—C19—H19	119.5
C8—C7—H7A	109.4	C18—C19—H19	119.5
C6—C7—H7B	109.4	C21—C20—C19	119.83 (6)
C8—C7—H7B	109.4	C21—C20—H20	120.1
H7A—C7—H7B	108.0	C19—C20—H20	120.1
C7—C8—C1	111.89 (4)	C22—C21—C20	119.68 (6)
C7—C8—H8A	109.2	C22—C21—H21	120.2
C1—C8—H8A	109.2	C20—C21—H21	120.2
C7—C8—H8B	109.2	C21—C22—C23	120.25 (6)
C1—C8—H8B	109.2	C21—C22—H22	119.9
H8A—C8—H8B	107.9	C23—C22—H22	119.9
C5—N9—C1	109.70 (4)	C22—C23—C18	120.58 (6)
C5—N9—C17	110.82 (4)	C22—C23—H23	119.7
C1—N9—C17	114.54 (4)	C18—C23—H23	119.7
O10—C10—C11	112.85 (5)		

### Hydrogen-bond geometry (Å, º)

**Table:**

*D*—H···*A*	*D*—H	H···*A*	*D*···*A*	*D*—H···*A*
O10—H10*A*···O3^i^	0.84	2.11	2.9298 (9)	165

Symmetry code: (i) −*x*+1, −*y*+1, −*z*.
